# Breast cancer survival experiences at a tertiary hospital in sub-Saharan Africa: a cohort study

**DOI:** 10.1186/s12957-015-0632-4

**Published:** 2015-07-19

**Authors:** Moses Galukande, Henry Wabinga, Florence Mirembe

**Affiliations:** Department of Surgery, College of Health Sciences, Makerere University, P. O. Box 7072, Mulago Hill Road, Kampala, Uganda; Department of Pathology, College of Health Sciences, Makerere University, P. O. Box 7072, Kampala, Uganda; Department of Obstetrics and Gynaecology, College of Health Sciences, Makerere University, P. O. Box 7072, Kampala, Uganda

**Keywords:** Breast cancer, Survival, Low-income country, Uganda

## Abstract

**Background:**

Cancer of the breast is a major health burden and the most common cancer among women worldwide. Though its incidence is fourfold greater in high-income countries, in sharp contrast, mortality rates are greatest among the low-income countries. Early detection linked to appropriate treatment is the most effective strategy to improve survival. The purpose of this study therefore was to establish the survival experiences of women with breast cancer at a Ugandan hospital.

**Methods:**

This study is an observational analytical study. It involved 262 women during the periods 2004 to 2007 and 2010 to 2012. Kaplan Meier method and Cox regression were used to calculate breast cancer mortality and cumulative survival experiences.

**Results:**

Sixty-three out of 262 (23 %) deaths were observed; mean age was 45 years, and 91 observations ended on or before follow-up. Luminal B median survival was months. The 5-year cumulative survival was 51.8 %. There were no stage I and II deaths. There were no differences in survival by phenotype adjusted for age, but there were differences for stage IV (*p* = 0.05).

**Conclusions:**

The cumulative 5-year survival was 51.8 %. The burden of advanced disease and associated mortality were high, and a significant number of patients were lost to follow-up after their first contact.

## Background

Breast cancer remains the most common cancer in women globally and only second to cervical cancer among Ugandan women (excluding HIV-related Kaposi’s sarcoma) [1]. It is estimated that worldwide, over half a million women died in 2011 due to breast cancer [2]. Although breast cancer was previously thought to be a disease of the developed world, almost 50 % of breast cancer cases and 58 % of deaths occur in less developed countries [1].

Breast cancer among Ugandan women as is seen among other black women in North America and Europe is characterized by poor survival experiences, aggressive behavior, and late stage at presentation, and a significant population is found among young women less than 40 years of age [3–6].

The reasons for these disparities in epidemiology and tumor behavior are a subject of much speculation and interest [3, 7, 8]. Breast cancer survival experiences vary greatly worldwide, ranging from 80 % or over (cumulative 5-year survival) in North America, Sweden, and Japan to around 60 % in middle-income countries and below 40 % in low-income countries [9]. Early detection linked to appropriate treatment is currently the most effective strategy to reduce breast cancer mortality. The low survival in less developed countries can be explained mainly by the lack of early detection programs and awareness resulting in a high percentage of women presenting with late stage disease, as well as lack of adequate treatment facilities [10, 11]. Whereas we now know that breast cancer has different subtypes (categorized by hormonal receptor status) and furthermore know that the prognosis for the different subtypes is different, we have not at all or conclusively examined survival by molecular subtypes in Uganda and the East African region. In addition, there is paucity of published data on breast cancer survival experiences in Africa in general and the East African region in particular.

In this study, we examined breast cancer survival experiences among a cohort of Ugandan women at a tertiary referral hospital.

## Methods

### Study design

This study is an observational analytical study.

### Study site

Mulago Hospital where this study was conducted is one of the two national referral hospitals in Uganda with a 1500-bed capacity and with over 400 physicians. The Ugandan Cancer Institute (UCI) housed on the same campus as Mulago Hospital is the only specialized public cancer treatment center in the country.

### Sample collection

Patients with histological diagnosed breast cancer during the period 2004–2007 and 2010–2012 were sampled for inclusion in the study. The case definition was a histologically confirmed invasive breast cancer in female patients.

Formalin-fixed and paraffin-embedded blocks for the 2004–2007 participants were retrieved from the archives in the Department of Pathology College of Health Sciences, Makerere. The department serves both Mulago Hospital and the UCI and matched them with corresponding demographic and clinical data from UCI records department.

Patients with non-available clinical data were excluded. The 2010–2012 participants were recruited prospectively, and the tissue samples were analyzed as soon as they were obtained.

### Inclusion and exclusion criteria

Patients with single primary, incident, invasive breast cancer diagnosed within the specified consecutive calendar period with a potential follow-up period of 2–5 years were included. Duplicate cases were excluded.

The period between 2008 and 2009 had significant missing files and specimen so it was excluded from what would have been a 2004–12 study period.

### Follow-up

A follow-up period of 2 years for the 2010–12 group and of 5 years for 2004–7 was imposed. Complete follow-up was achieved when vital status (alive/dead) at the applicable closing date was known for an individual. If not known, then the follow-up was incomplete.

We employed predominantly active follow-up methods. Information on deaths was sourced from patient clinical data files for scheduled and unscheduled return visits. This included repeated periodic scrutiny of medical records at UCI and the breast unit in Mulago, visits to Hospice Uganda, and enquiries with attending physicians. In addition, telephone enquiries from patients or persons known to them were made, and we also checked at the Makerere University cancer registry database.

### Censoring

With a closing date of follow-up for the subjects that dropped out or lost before the closing period were considered censored. When the loss was due to a factor unrelated to the study outcome, it was termed random (non-informative censoring). When it occurred due to a factor related to the study outcome death, then it was considered non-random or informative censoring.

The Cox model was used, and the determinants tested for association with loss to follow-up were age at diagnosis and stage of disease.

### Index date and closing date to follow-up

The index date is the starting date for calculation of survival, and this was the first date of unequivocal diagnosis of cancer by means of histological diagnosis. The inclusion dates were between the 1st of January 2004 to the 31st of December 2007 and the 1st of January 2011 to the 31st of December 2012 with a closing date on the 1st of January 2009 to the 31st of December 2014, respectively.

### Survival time

Survival time was calculated at the time (in months or completed years) between the index date and the date of death, date of loss to follow-up, or the closing date, whichever was earliest.

### Other variables

Age at diagnosis was defined as the age in completed years on the incidence date, and age was not necessarily verified with birth certificates.

Clinical extent of disease: Manchester staging was used, stages I, II, III, and IV:

Stage I. Growing but localized disease only
Stage II. Growing localized disease ± nodal involvement
Stage III. Growing locally advanced disease (involving chest wall, skin) ± nodal involvement
Stage IV. Metastatic disease

In the 2004–7 period, 383 files were retrieved; 213 were excluded either because pathology blocks could not be identified or clinical notes were grossly insufficient, leaving 170 files. Fifty-four of these were excluded either because the histopathological analyses were inconclusive for breast cancer or outright benign, leaving 116 data that were included in the study.

**Fig. 1:**
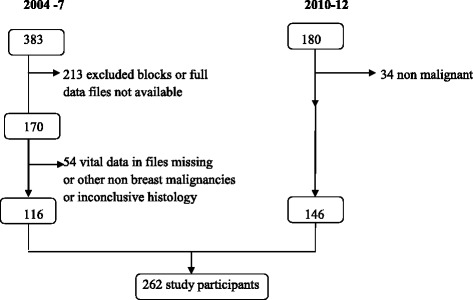
Patient recruitment chart from the 2004–2007 and 2010–12 cohorts

In the 2010–12 period, 180 participants data were considered, 34 were non-malignant and were excluded, and 146 were included in the study (see Fig. 1).

### Data quality indices

All cases had histologically confirmed breast cancer diagnosis. The cases registered based on death certification only were nil.

### Statistical analysis: estimation of survival probability

We used life tables Kaplan Meier and Cox regression as the tool to describe the mortality experience of this hospital-based case series.

We chose these methods of estimating survival probability because they handle censoring by assuming it to be random. They permitted the calculation of the cumulative of survival at time from the conditional probabilities of survival during intervals of follow-up time. The number of cases censored during the interval, because of loss to follow-up or withdrawal, was shown.

Subjects who experienced the outcome during each interval were indicated. The effective number of subjects at risk during each interval was calculated.

The starting point was defined as the date of the first diagnosis; the outcome of interest was death. The end point was a binary variable (alive or dead). The calculation of survival time involved patient accrual period during which patients were recruited and followed up, and closing date for analysis was defined as 2–5 years.

The probability (risk) of dying was calculated by year 5. Cumulative survival probabilities were calculated separately for stage, age, and phenotype. Statistical tests for formal comparison of the different survival curves were done by log rank test to assess for statistical significance of differences observed.

All data were analyzed using STATA version 12.

### Ethical consideration

The study was approved by the Makerere University College of Health Sciences School of Medicine Research and Ethics Committee and was registered with Uganda National Council of Science and Technology.

### Evaluation of ER, PR, and HER2 status and quality assurance

We used the quality assurance guidelines of the College of American Pathologists (CAP). The laboratory had control tissue, which has proven positive for each of the antibodies; a section of the positive control was used at every run of the day, and a negative control was run.

Paraffin specimens were cut into four sections and mounted on positively charged slides. The slides were paraffinized and rehydrated in xylene followed by graded alcohols, then washed in Tris-buffered saline. The immunohistolochemical assays were performed using an immunostainer with antibodies and antigen unmasking.

Appropriate negative controls for the immunostaining were prepared by omitting the primary antibody step. The results were scored semi quantitatively using Reiner’s four-point scale based on intensity and percentage of IHC reaction; HER2 staining were evaluated according to manufacturer’s instructions [12].

Antibodies used were the following: ER (clone SP-I), ASR PR (clone Y85), ASR and HER2/neu (c-erbB-2), and clone CB-11). The manufacturer for all was Cell marquee corporation, Rocklin, CA 95677.

### Evaluation of histopathological features

Hematoxylin and eosin (H&E) staining was performed first to confirm diagnosis of invasive breast cancer before immunostaining. The histological type and grade were determined. A consultant pathologist(s) and laboratory technicians received all the histological slides, and the tumors were classified according to Nottingham modification of the Scorff Bloom Richardson criteria [13]. Based on histology, tumors were classified into the following groups: invasive ductal carcinoma (NoS), lobular, medullary, papillary, and colloid.

## Results

Data from a cohort of 262 women diagnosed with breast cancer at Mulago Hospital and UCI during the years 2004–7 and 2010–12 and followed up from the point of diagnosis were included in assessment of their survival experience.

Sixty-three events were recorded, 56 with specified sub types, and 7 with undetermined subtypes; 91 participants made only one contact visit to the health facility.

The mean age of the study participants was 45 years 6 months. The majority were stage III and IV 187/262 (71 %). Subtypes triple-negative breast cancer (TNBC) and HER2+ contributed 124/243 (51 %) of verified subtypes. The mean age of observed events (deaths) was 45.3 years. There were no deaths recorded for stage I and II disease (see Table 1).

**Table 1:** Characteristics of breast cancer survival study participants, Uganda 2014

Variable	Number
Overall mean age	45 years 6 months
Stage	
I	5
II	17
III	152
IV	35
Inconclusive or missing	53
Sub types	
TNBC	83
Luminal A	93
Luminal B	26
HER2+	41
Unspecified or missing	19
Cumulative survival	0.518
Observed events (death)	
Mean age	45.3 years
Deaths	
≥40 years	32 (50.1 %)
≤40 years	31 (49.9 %)
TNBC	
I	0
II	0
III	14
IV	3
Unspecified or missing	1
Luminal A	
I	0
II	0
III	17
IV	6
Unspecified or missing	2
Luminal B	
I	0
II	0
III	4
IV	4
Unspecified or missing	0
HER2+	
I	0
II	–
III	6
IV	0
Unspecified or missing	
I	–
II	0
III	1
Total	
I	0
II	0
III	42
IV	13
Unspecified or missing	3

The luminal B group had median survival time of 28 months, and the rest did not reach the 50 % with probability of failure (death) on the KM curve. However, 25 % of subjects in TNBC group failed by 16 months, in luminal A were 18 months, luminal B 14 months, and HER2+ by 36 months (see Table 2).

**Table 2:** Cumulative survival probabilities by subtypes, a breast cancer study, 2014

	Time	Survivor function	Std. error	[95 % Conf. int.]
TNBC	0	1	–	–
	12	0.837	0.053	0.6994 0.9153
	24	0.6647	0.0719	0.5034 0.7843
	36	0.6647	0.0719	0.5034 0.7843
	48	0.6136	0.0826	0.4325 0.7523
	60	0.4832	0.1048	0.2711 0.6668
Luminal A	0	1	–	–
	12	0.8355	0.0477	0.7155 0.9081
	24	0.6593	0.0622	0.5223 0.7655
	36	0.5539	0.0719	0.4031 0.6810
	48	0.5113	0.078	0.3509 0.6507
	60	0.4601	0.0853	0.2895 0.6150
Luminal B	0	1	–	–
	12	0.8125	0.0976	0.5246 0.9354
	24	0.5909	0.1302	0.3026 0.7933
	36	0.3152	0.1623	0.0628 0.6177
	48	0.3152	0.1623	0.0628 0.6177
	60	0.3152	0.1623	0.0628 0.6177
Her2+	0	1	–	–
	12	0.92	0.0543	0.7164 0.9794
	24	0.805	0.0897	0.5524 0.9238
	36	0.7245	0.1111	0.4409 0.8809
	48	0.7245	0.1111	0.4409 0.8809
	60	0.4528	0.1695	0.1353 0.7307

### The probability of death by stage

**Fig. 2:**
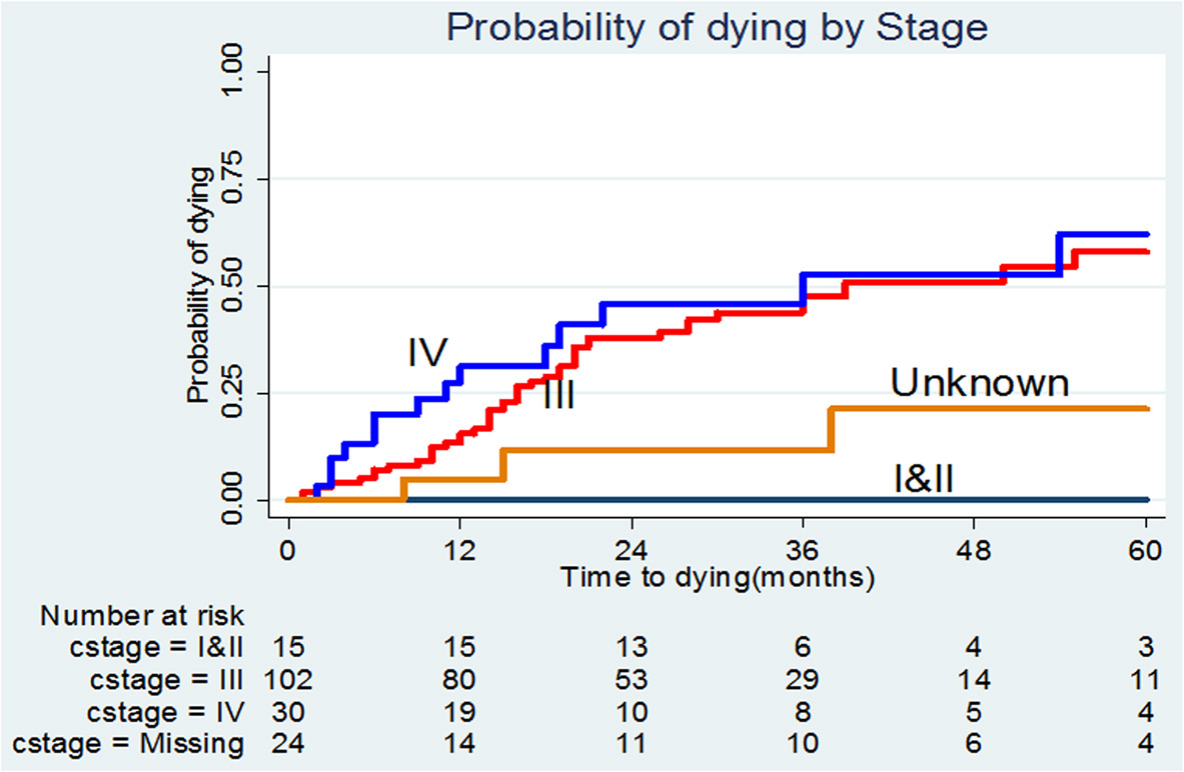
Probability of death by stage

The differences in probability of dying by stage (hazard) were significant *p* < 0.001 (Cox Breslow method for ties). The odds of dying were higher for stage IV disease followed by stage III (see Fig. 2).

In Fig. 2, the “unknown” curve represents missing or inconclusive staging.

**Fig. 3:**
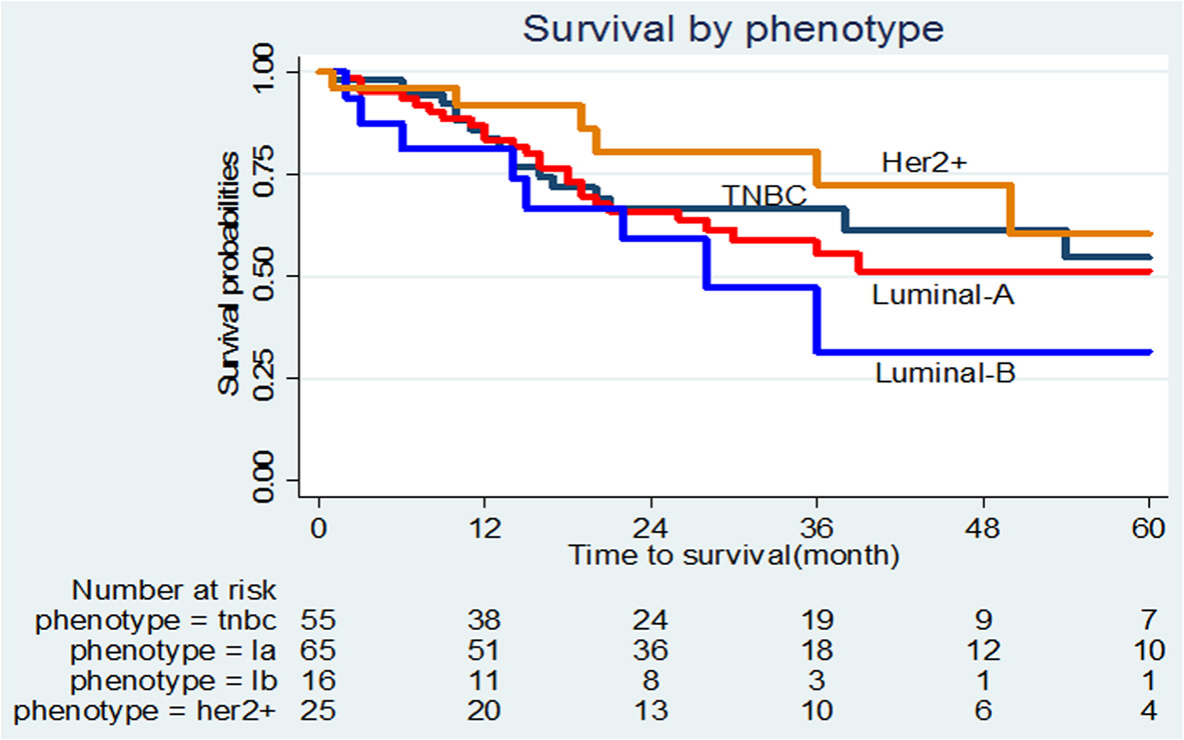
Survival by phenotype

**Fig. 4:**
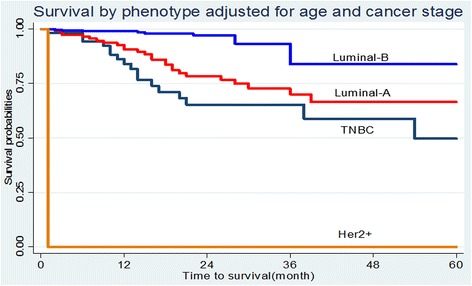
Survival by stage-adjusted for age and phenotype

In Fig. 3, survival by phenotype is demonstrated *p* = 0.2941. Her2+ and TNBC show better survival probabilities, however, when adjusted for age and stage (see Fig. 4). HER2+ and TNBC showed the lowest survival probabilities.

The stage III and IV survival probabilities were less than 0.4 at 5 years (60 months) (see Table 3).

**Table 3:** Summarizes the survival function of the different stages, a breast cancer study, 2014

Time (months)	I and II			III			IV			Missing/inconclusive		
	Survival probability			Survival probability			Survival probability			Survival probability		
	95 % CI			95 % CI			95 % CI			95 % CI		
0	1			1			1			1		
12	1			0.8446	0.7553	0.9033	0.689	0.4859	0.8251	0.9524	0.7072	0.9932
24	1			0.6204	0.5124	0.7111	0.5414	0.3269	0.714	0.8844	0.6029	0.9706
36	1			0.5236	0.4073	0.6274	0.4737	0.2546	0.6649	0.8844	0.6029	0.9706
48	1			0.4909	0.3653	0.6048	0.4737	0.2546	0.6649	0.7861	0.4492	0.9302
60	1			0.3442	0.2029	0.4902	0.3789	0.1562	0.6022	0.6289	0.2372	0.8611

## Discussion

Clinical advances in breast cancer treatment during the past five decades have led to major improvement in health outcomes including longer disease free survival, less surgical mutilation, and increasing individualization of treatment [14–16]. In addition to improved early detection, successful treatment of regional disease with nodal involvement has also made some dent on late stage mortality [14]. These facts hold true mostly for high-income countries. In upper and middle-income countries, five-year survival rates are consistently over 80 %, in sharp contrast, survival falls as low as 12 % in low-income countries such as in Gambia [17]. A recent population-based study put the 5-year survival in Uganda at 44 % [18].

The possible reasons for the poorer survival rates in low-income countries are varied and include but not limited to the following: delayed individual health seeking behavior, low socio economic circumstances, limitations and inadequacies in health systems, and a lack of prioritization for non communicable diseases [10, 11, 19, 20]. And more specifically, lack of capacity to do early detection as well as adequate diagnostic and treatment facilities [21, 22].

In a recent Ugandan study [19], women delayed up to 120 months before seeking appropriate breast cancer care with such excessive delays play a part in allowing tumors to advance in stage. In the same study, 89 % were stage III and IV (late) stage disease [19].

Participants with stage I and II had survival probabilities of 1 (100 %), and no death was recorded in this category over the 5-year period. This matches or nearly matches the experiences recorded in the high-resourced settings where a 10-year survival probability is equal or more than 97 % for stage I and II disease [9].

The survival for early disease was high despite all the health service delivery limitations. This implies that we do have an opportunity to improve survival only if we make early diagnoses. We therefore recommend urgent and considerable investment in understanding the drivers of late disease presentation and encourage women to report symptoms at the earliest opportunity but also for the women to demand for appropriate screening regularly.

The cumulative survival after 5 years in this study was 51.8 %, a little higher than what is shown for low-income countries. Although with these data, the overall median survival rate was not reached; only the median survival for luminal B sub type was determined which was 28 months. To reach the median survival rate, more time would be needed to accumulate 50 % events (probability in this case was death). This was a hospital-based case series strictly speaking not comparable to population-based studies. This rate 51.8 % could have been an overestimate given the number of patients censored.

Half of the women who died were 40 years old or younger with a mean age of 45 years. This adds to the characterization of breast cancer disease landscape in Uganda a low-income country in sub-Saharan Africa. The majority (89 %) was stage III and IV. Stage IV disease is metastatic and patients normally die from complications of metastases. In this study, the majority (of those) who died were stage III. This stage was not revised at the time of death so it is likely that most if not all had advanced to stage IV disease. The staging processes in this context mostly involved a plain chest X-ray, abdomino-pelvic ultra sound scanning. In some few cases CT-scan, MRI scan, or bone scans were used. The latter poses better precision but are mostly inaccessible in the low resourced environments, like this one. With inadequate high-precision staging capacity, the tumor spread (stage) at diagnosis was likely to be under assessed.

This study revealed that more than a third of women (35 %) with breast cancer were lost to follow-up under one year, after only one contact with the facility. This could be due to several factors (though this study did not investigate them). Some could have died, others could have sought other opinions elsewhere especially from alternate medicine sources (herbal, spiritual or traditional healers). It is also possible that they found considerable challenges in navigating a complex health care system, which does not have dedicated patient navigators to help patients work their way through it. The majority of these patients have low level formal education and may be unfamiliar with dealings in an urban hospital setting. Irrespective of the reasons, this was a significant loss to follow-up that warrants specific attention to mitigate further losses in the future. These losses imply impeded access (barriers) to appropriate and or prompt cancer care. These impediments need to be tackled urgently. Forty percent had TNBC and HER2+ cancer phenotypes; these phenotypes carried the lowest survival rates in this study. These were ER-negative tumors and not amenable to anti-hormonal therapy. For TNBC and HER2+, targeted therapies are recommended but normally not affordable to most patients, since the public service often does not stock them.

Significant challenges are experienced in studies that involve follow-up of patients for long periods of time especially in our context. They include but not limited to a lack of identifiable physical address, reliable phone contacts, high rate of migration from place to place, traveling long distances, mistrust in health care system, availability and use of unlicensed practitioners (traditional healers) who normally keep no records and do not communicate with the main stream health care system [22, 23]. Secure record storage and accurate recording are also considerable challenges for field studies. These barriers put together make it impossible for the majority of our patients to have the best possible chance to survive their own experience of this disease.

### Study limitations and strengths

Although the date of onset of the disease would seem more appropriate for defining the start of counting survival time, the date for first diagnosis is used in such studies. It is generally observed that the time lag between onset of symptoms and presenting to hospital for a diagnosis to be made may be long, therefore leading to an under estimate in survival measuring.

A possibility of under reporting deaths from other causes may overestimate the cause specific survival probabilities. It was difficult to establish whether the cause of death was unrelated to breast cancer, as a significant proportion of patients died at home, and in addition, post mortems were not commonly done.

On the other hand, this study could have overestimated cumulative survival due to a large number of censored patients. Although an additional sense of poor survival was given by the low number of patients at the end of the follow-up period.

Although cancer survival estimated from hospitals case series at best reflects experiences of the selective groups of patients in specific settings they cannot be generalized as reflecting the overall efficiency of the cancer health services in a given country or region. In this study, our findings reflect the enormous challenges faced in initiating and completing treatment.

A sub analysis of survival by treatment type and completion was not done.

## Conclusions

Breast cancer survival was low, most patients had late stage disease at presentation, a significant number did not complete care (treatment), and many dropped out before the first year was completed. TNBC and HER2+ carried the lowest survival rates. These data emphasize the need for urgent and adequate investment in patient support to complete treatment and follow-up. Further exploration of the relationship between survival and breast cancer molecular subtypes in the sub Saharan setting is recommended.

## Abbreviations

BSE: breast self examination
CBE: clinical breast examination
TNBC: triple-negative breast cancer
